# The Effectiveness of Interactive Text Messaging and Structured Psychosocial Support Groups on Developmental Milestones of Children From Adolescent Pregnancies in Kenya: Quasi-Experimental Study

**DOI:** 10.2196/37359

**Published:** 2023-05-01

**Authors:** Valerian Mwenda, Ireen Makena, Vincent Ogweno, James Obonyo, Vincent Were

**Affiliations:** 1 Department of Non-communicable Diseases Ministry of Health Nairobi Kenya; 2 Field Epidemiology and Laboratory Training Program Ministry of Health Nairobi Kenya; 3 Field Epidemiology Society of Kenya Nairobi Kenya; 4 Department of Biological Sciences Chuka University Chuka Kenya; 5 Department of Pediatrics University of Nairobi NAIROBI Kenya; 6 County Department of Health Homa Bay County Homa Bay Kenya; 7 Kenya Medical Research Institute-Wellcome trust Nairobi Kenya

**Keywords:** text messages, adolescent pregnancy, milestones, mHealth, psychosocial support, Kenya, nurturing care

## Abstract

**Background:**

In sub-Saharan Africa, one-quarter of all pregnancies occur in adolescents. Children born to adolescent mothers have poorer physical and socio-cognitive development. One reason may be inadequate knowledge on childcare and psychosocial support during pregnancy and post partum, since adolescent mothers have less antenatal care attendance and overall interaction with the health care system. Mobile health technology has been used to relay health information to special groups; however, psychosocial support commonly requires physical interaction.

**Objective:**

We aimed to assess the efficacy of an interactive mobile text messaging platform and support groups in improving adolescent mothers’ knowledge and practices as well as infant growth and development.

**Methods:**

This was a quasi-experimental study, conducted among adolescent mothers with infants younger than 3 months, in Homa Bay County, Kenya. Five of the 8 subcounties in Homa Bay County were purposively selected as study clusters. Four subcounties were assigned as intervention clusters and 1 as a control cluster. Adolescent mothers from 2 intervention subcounties received interactive text messaging only (limited package), whereas those from the other 2 subcounties received text messaging and weekly support groups, moderated by a community health extension worker and a counselor (full package); the control cluster only received the end-line evaluation (posttest-only control). The follow-up period was 9 months. Key outcomes were maternal knowledge on childcare and infant development milestones assessed using the Developmental Milestones Checklist (DMC III). Knowledge and DMC III scores were compared between the intervention and control groups, as well as between the 2 intervention groups.

**Results:**

We recruited 791 mother-infant pairs into the intervention groups (full package: n=375; limited package: n=416) at baseline and 220 controls at end line. Attrition from the intervention groups was 15.8% (125/791). Compared with the control group, adolescent mothers receiving the full package had a higher knowledge score on infant care and development (9.02 vs 8.01; *P*<.001) and higher exclusive breastfeeding rates (238/375, 63.5% vs 112/220, 50.9%; *P*=.004), and their infants had higher average DMC III scores (53.09 vs 48.59; *P*=.01). The limited package group also had higher knowledge score than the control group (8.73 vs 8.01; *P*<.001); this group performed better than the full package group on exclusive breastfeeding (297/416, 71.4% vs 112/220, 50.9%; *P*<.001) and DMC III scores (58.29 vs 48.59; *P*<.001) when compared with the control group. We found a marginal difference in knowledge scores between full and limited package groups (9.02 vs 8.73; *P*=.048) but no difference in DMC III scores between the 2 groups (53.09 vs 58.29; *P*>.99).

**Conclusions:**

An interactive text messaging platform improved adolescent mothers’ knowledge on nurturing infant care and the development of their children, even without physical support groups. Such platforms offer a convenient avenue for providing reproductive health information to adolescents.

**Trial Registration:**

Pan African Clinical Trials Registry PACTR201806003369302; https://tinyurl.com/kkxvzjse

## Introduction

The World Health Organization (WHO) estimates that 23 million girls younger than 20 years of age become pregnant in low- and middle-income countries (LMICs) every year [1]. The burden of adolescent pregnancies is the highest in Sub-Saharan Africa, where one-quarter of all pregnancies occur in adolescents [2]. In Kenya, the adolescent pregnancy rate is 96 per 1000 births, with some counties having a higher burden [3].

Adolescent pregnancies are associated with poorer physical and socio-cognitive development during infancy and early childhood, partly due to inadequate knowledge on infant care [1]. Children born to mothers younger than 20 years of age have a 50% higher chance of being born as stillbirths, dying within the first few weeks after birth or being born with low birth weight; they also have a higher risk of long-term effects, behavioral problems in childhood, poor cognitive development, and worse educational outcomes [4-9]. Adolescent mothers have higher rates of depression, poverty, and poor economic development since the majority are forced to drop out of school [5,10].

Adolescent mothers show less sensitive and more intrusive, hostile interactive behaviors and less frequently engage in direct interactions with their children [11]. Maternal sensitivity has also been shown to be of major significance for children’s attachment and socio-emotional development as well as cognitive development [12,13]. Another study showed that cognitive developmental differences in 3-year-old children of adolescent and adult mothers were indirectly mediated by maternal parenting behaviors [14]. Some of these behaviors among adolescent mothers may arise from the lack of knowledge on proper infant feeding and care practices. Socioeconomic problems such as economic deprivation, which accompany many adolescent pregnancies, are associated with negative developmental outcome in the offspring [15]. This may be due to low cognitive stimulation in the home, including toys, books, and learning opportunities that shape the developing brain [16].

Adolescent mothers have less knowledge on infant care and development compared with their older counterparts [17,18]. Antenatal care (ANC) offers an opportunity for the provision of information and advice for a healthy pregnancy, safe childbirth, and postnatal recovery; care of the newborn; and promotion of early, exclusive breastfeeding [19]. However, adolescent mothers also attend ANC less often than recommended [20]. Therefore, a different approach is necessary for ensuring that these mothers receive this vital information. Identifying adolescent pregnancies early on and offering a specialized package of care involving education, counseling, and support together with nutritional supplementation can reduce neurocognitive underdevelopment at minimal cost in LMICs [21,22]. Mobile health (mHealth) technology has been applied in various settings in LMICs to motivate positive health behaviors and improve health outcomes, including HIV testing [23], antiretroviral treatment adherence [24-26], postoperative clinic follow-ups [27], malaria treatment [28], immunization [29], and postnatal care and follow-ups [30]. Such innovations have been shown to be cost-effective in improving maternal, newborn, and child health outcomes [31,32].

The WHO Nurturing Care Framework identifies the formation of parent groups, counseling, and support as a model for achieving responsive care giving [33]. We sought to test the efficacy of interactive mobile text messaging on childcare and development, with or without psychosocial support groups, among adolescent mothers in improving maternal knowledge and early infant development.

## Methods

### Study Design and Population

This was a quasi-experimental study to assess the efficacy of a nurturing care package delivered through mHealth and psychosocial support on the development of children born to adolescent mothers. The study was conducted in 5 subcounties purposively selected from the 8 subcounties in Homa Bay County, Kenya. Homa Bay County has the second highest prevalence of adolescent pregnancies in Kenya, at 33.3% in 2019 [34].

The study population was adolescent mothers and their infants. The minimum sample size required per cluster was 200, based on a 22% expected difference in exclusive breastfeeding rates between adolescent and mature mothers [35]. Adolescent mothers (aged younger than 20 years) with a child younger than 3 months of age at recruitment, who are residents of 1 of the selected subcounties, were eligible for inclusion. We excluded those who did not have access to a mobile phone within the family setup. Written consent was obtained from all the participants; for those younger than 18 years of age, a guardian had to give consent before inclusion.

### Sampling Procedures

**Figure 1:**
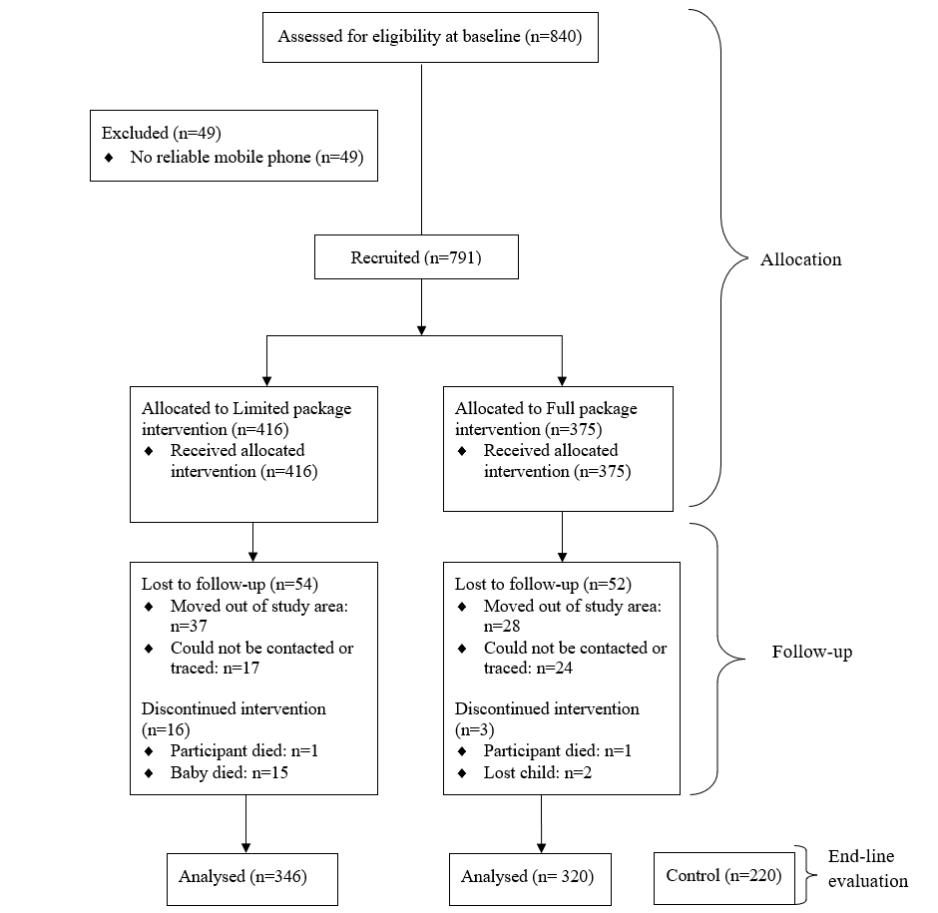
Study CONSORT (Consolidated Standards of Reporting Trials) flow diagram.

We adopted a cluster-sampling method, with the subcounty representing a cluster. Four subcounties were randomly selected from the 8 in Homa Bay County and assigned to 1 of the 2 study arms: full package intervention or limited package intervention (2 subcounties each). Eligible adolescent mothers were recruited by community health extension workers working in each of the subcounties. A baseline survey was conducted, collecting key variables including age, education, occupation, telephone number, and knowledge and practices on infant care and development. A total of 791 adolescent mothers were recruited into the study at baseline. We did not recruit a no-intervention control group at baseline due to 2 reasons. First, the community health extension workers in the study locations felt that recruiting a control group and then following up with them until the final evaluation with no intervention may be difficult to explain to the adolescents and their guardians. On the contrary, a control group at end line would be packaged as a health promotion activity in the community. Second, since the study team would still need to do community tracking of the adolescents in the control group, it would be difficult to avoid providing information similar to our interventions if specifically asked by the participants; this would result in contamination risk. Therefore, we adopted a pretest-posttest design with a posttest-only control [36]. We recruited a control group with comparable key characteristics (maternal age younger than 20 years with a child between the age of 9-12 months) from a different subcounty in Homa Bay County at end line. A total of 220 adolescent mothers were recruited in the control group (Figure 1).

### Intervention

The intervention consisted of (1) the delivery of targeted messages on childcare and nurturing through an interactive text messaging platform and (2) psychosocial support groups for the adolescent mothers, moderated by trained personnel. This was based on the WHO Nurturing Care Framework, which emphasizes health, nutrition, early learning, and responsive care giving through parent groups, counseling, and support [33]. By delivering a package of targeted interventions to the adolescent mothers, we expected to improve their knowledge and practices in childcare, which would translate to better development of their children. We hypothesized that although an mHealth platform could provide an avenue for health education, psychosocial support for the adolescent mothers may still need a physical interaction. The interventions were implemented in 4 subcounties: 2 received text messaging only and 2 received the full package of text messaging and psychosocial support groups. The text messaging intervention consisted of the transmission of text messages, arranged in themes each week, at a rate of 5-10 key messages per week. These themes included feeding at every age (including during diarrhea and other illnesses), breastfeeding, immunization, danger signs in infants and young children, general childcare and safety, developmental milestones, and when to seek care. The interactive feature allowed the adolescent mothers to engage experts on the platform to seek clarification, ask questions, or obtain guidance on various aspects of their health or that of their children, at no cost on their side. The psychosocial support groups consisted of 5-10 adolescent mothers, grouped by the ward of residence. Each group held weekly meetings, with a community health extension worker and a counselor moderating the discussions. Discussion topics were based on the themes addressed by the text messages transmitted that week. Meetings were usually held during weekends, to accommodate adolescent mothers who were in school. The total follow-up period was 9 months; the interventions were conducted from October to December 2019, and the postintervention follow-up was conducted from January to June 2020.

### Data Collection

A baseline questionnaire was administered, documenting the demographic and contact information of the eligible participants. Key information at baseline included the ages of the mother and infant, residence, and mobile telephone number. Data were collected by community health extension workers. Development assessment used the Developmental Milestones Checklist (DMC III) [37], with questions from the checklist matched with the age of the infant at final evaluation. The DMC III items had 3 likely responses: milestone observed continually (score 2), milestone observed occasionally (score 1), and milestone not observed at all (score 0). The DMC III evaluation involves both interviewing the mother as well as observing and assessing the infant. Data were collected electronically using Android tablets. A survey questionnaire was administered to the adolescent mothers at end line, measuring knowledge (12 items) and practices in feeding, vaccination, growth monitoring, breastfeeding, hygiene, and milestones in a child’s development. Each of the survey questions was scored as either 0 if the mother did not know the correct response or 1 for correct responses. For the DMC III checklist, the gross motor component had 25 items, the fine motor component had 12 items, and the language component had 18 items. Each item was either scored 0 (caregiver has not observed that milestone in the previous 4 weeks), 1 (the milestone item has been observed but not continuously), or 2 (the milestone had been observed continuously in the previous 4 weeks). In addition to the questionnaire administered to the mother, other assessments on the infants included weight, midupper arm circumference, head circumference, and height or length.

### Study Outcomes

The primary outcomes were (1) maternal knowledge and practices (exclusive breastfeeding, immunization, feeding, and stimulation) on childcare and development and (2) infant developmental milestones at end line as assessed by the DMC III. The secondary outcomes were (1) the incidence of diarrhea and respiratory illnesses and (2) anthropometric measurements. The secondary outcomes are not reported in this paper.

### Data Management and Statistical Analysis

We first explored the descriptive analysis using independent sample 2-tailed *t* test to compare mean estimates of the outcomes between study arms and by survey period. The study end points were analyzed at end line only, comparing the 2 interventions against the control, as well as against each other (full package vs control, limited package vs control, and full package vs limited package). Development milestones were summed to generate continuous variables representing knowledge, fine motor, gross motor, and language scores. These were considered normally distributed variables, and hence, means and SDs were used to compare the significant differences. Independent sample *t* tests were then used to test the hypothesis that the mean differences in DMC III scores were similar for those participants who received the full package intervention compared to controls. To account for cluster effects and probability sampling weights and to assess the effectiveness of the interventions at the same time, we fitted a generalized estimating equations (GEE) model with a linear model for a continuous outcome. The GEE model was used with a small-sample correction, due to the small number of clusters [38]. The dependent variables were knowledge, language, gross motor, and fine motor scores. The effect factor was a binary variable representing either full package versus control, limited package versus control, or full package versus limited package. Results with *P* values <.05 were considered statistically significant results. The analysis was done using Stata (version 15; StataCorp), and effects were reported with 95% CIs. Data management was carried out by a statistical team that was independent from the main study group.

### Ethics Approval

Eligible study participants were given the rationale and aims of the intervention study. Written informed consent was then obtained from all enrolled adolescent mothers. The protocol was approved by the African Medical Research Foundation Ethics and Scientific Review Committee (protocol ESRC P589/2019). We also obtained approval of local authorities, at the county and subcounty levels, and community strategy coordination before the implementation of the study. The study protocol was registered at the Pan African Clinical Trials Registry (PACTR201806003369302).

## Results

### Baseline Sociodemographic Characteristics of the Study Population

A total of 1011 adolescent mothers were recruited: 416 in the limited package arm, 375 in the full package arm, and 220 in the control group. The majority of the participants (873/1011, 86.4%) were between the ages 16 and 19 years, and 61.6% (623/1011) were students. Only 10% (101/1011) had completed secondary school. One-quarter (265/1011, 26.2%) were married, and only 16.4% (166/1011) were involved in any form of income-generating activity (business, employment, casual labor, or farming). Antenatal clinic attendance for at least 1 visit was high for all the groups (370/375, 98.7% for the full package group; 396/416, 95.2% for the limited package group; and 215/220, 97.7% for the control group). The comparison of the various parameters across the 3 groups is shown in Table 1.

At end line, 666 adolescent mothers in the intervention groups were still active in the study, translating to an overall attrition rate of 15.8% (125/791; 70/416, 16.8% in the limited package intervention group vs 55/375, 14.7% in the full package intervention group). The mean age of the children at the end-line evaluation was 11.8 (SD 2.5) months for the full intervention group, 12.4 (S.D 2.8) months for the limited intervention group, and 11.0 (S.D 4.2) months for the control group. Exclusive breastfeeding rates were higher in the full package intervention group compared with the control group (238/375, 63.5% vs 112/220, 50.9%; *P*=.004); the difference was even higher for the limited package intervention group (297/416, 71.4% vs 112/220, 50.9%; *P*<.001).

**Table 1:** Sociodemographic characteristics of study population^a^.

Characteristics			Full package (n=375), n (%)		Limited package (n=416), n (%)		Control (n=220), n (%)
**Age range (years)**							
	12-15	64 (20)		63 (15.1)		11 (5)	
	16-19	311 (82.9)		353 (84.9)		209 (95)	
**Education level**							
	Primary, incomplete	134 (38.7)		163 (39.2)		83 (37.5)	
	Primary, completed	38 (10.1)		59 (14.2)		27 (12.3)	
	Secondary, incomplete	156 (41.6)		162 (38.9)		88 (40.2)	
	Secondary, completed and above	47 (12.5)		32 (7.7)		22 (10.1)	
**Occupation**							
	Business	8 (2.1)		32 (7.7)		11 (5)	
	Casual laborer	6 (1.6)		7 (1.7)		4 (1.6)	
	Farming	24 (6.4)		50 (12)		20 (9.3)	
	Formal employment	0 (0)		2 (0.5)		0 (0)	
	Housewife	68 (18.1)		72 (17.3)		39 (17.9)	
	Student	260 (69.3)		227 (54.6)		136 (61.7)	
	Other	9 (2.4)		26 (6.3)		10 (4.5)	
**Marital status**							
	Married	84 (22.4)		123 (29.6)		58 (26.4)	
	Single	291 (77.6)		292 (70.2)		162 (73.6)	
	Other^b^ (separated, divorced, or widowed)	0 (0)		1 (0.2)		0 (0)	
**Source of family income**							
	Agriculture	128 (34.1)		286 (68.8)		115 (52.3)	
	Business	195 (52)		75 (18)		75 (34.1)	
	Employment	30 (8)		14 (3.4)		12 (5.6)	
	Others	22 (5.9)		41 (9.9)		18 (8)	
**Religion**							
	Christian	375 (100)		409 (98.3)		218 (99.1)	
	Muslim	0 (0)		7 (1.7)		2 (0.9)	
**ANC^c^ attendance at least 1 visit**							
	Yes	370 (98.7)		396 (95.2)		215 (97.7)	
	No	5 (1.3)		20 (4.8)		5 (2.3)	

^a^This includes the number of participants as at recruitment—the intervention groups at baseline and the control group at end line.

^b^Other includes those who are separated, divorced, or widowed; these were combined since the numbers were very small.

^c^ANC: antenatal care.

### Change in Maternal Knowledge on Infant Care and Developmental Milestones at End Line

Compared with the control group, adolescent mothers who received the full package had a higher knowledge score on infant care and development (9.02 vs 8.01; *P*<.001); the same was observed for those who received the limited package (8.73 vs 8.01; *P*<.001), as shown in Table 2.

**Figure 2:**
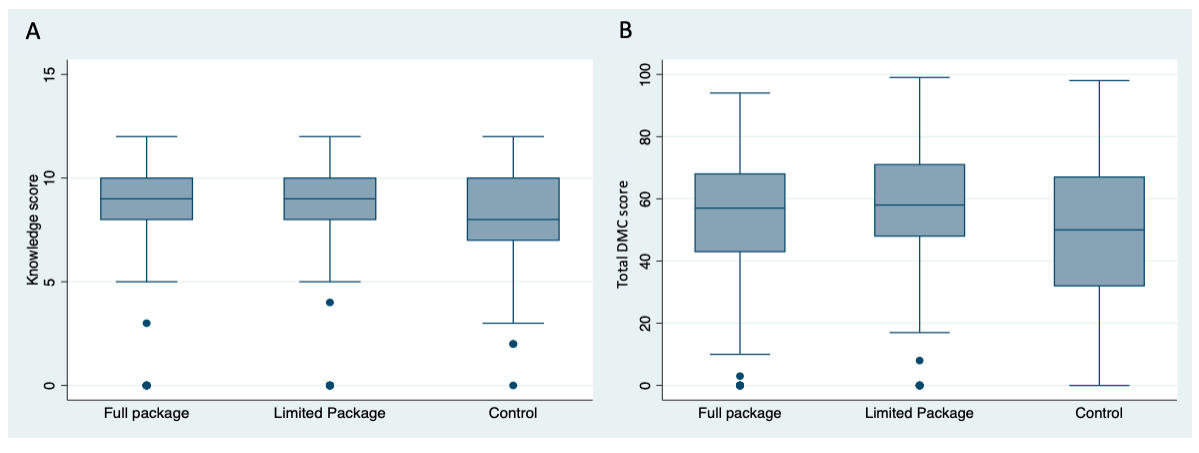
Comparison between groups in (A) maternal knowledge and (B) infant developmental milestones at end line. (A) Full package vs control: *P*<.001; limited package vs control: *P*<.001; full package vs limited package: *P*=.01. (B) Full package vs control: *P*=.01; limited package vs control: *P*<.001; full package vs limited package: *P*=.003. DMC: Developmental Milestones Checklist.

The infants of mothers in both intervention groups also had higher average scores on the DMC III in developmental milestones (Figure 2).

**Table 2:** Comparison of maternal knowledge score between the 2 interventions groups and the control group at end line^a^.

Package	Intervention			Control			Knowledge score, mean difference		*P* value (*t* test)
	Participant, n	Knowledge score, mean (SD)	Participant, n		Knowledge score, mean (SD)				
Full package	307	9.02 (2.19)	199		8.01 (2.07)	1.01		<.001^b^	
Limited package	297	8.73 (2.22)	199		8.01 (2.07)	0.73		<.001^b^	

^a^The n values differ from the numbers analyzed from the CONSORT (Consolidated Standards of Reporting Trials) diagram due to records with missing key variables that were dropped from the model.

^b^Significant results at *P*<.05.

### Differences in Developmental Milestones at End Line for the Intervention Groups and the Control Group

Compared with the control group, participants who received the full package had significantly higher gross motor (26.55 vs 23.50; *P*<.001), fine motor (12.97 vs 11.41; *P*<.001), as well as overall DMC III score (53.09 vs 48.59; *P*=.01). However, there was no significant difference in language scores between those who received the full package of interventions and the control group (13.57 vs 13.68; *P*=.56; Table 3).

Participants who received limited package also had significantly higher scores in fine motor (12.59 vs 11.41; *P*=.002), gross motor (27.95 vs 23.50; *P*<.001), and also language (17.75 vs 13.68; *P*<.001) scores compared with the control group. Overall DMC III scores were also significantly higher for those who received limited package compared with control (58.29 vs 48.59; *P*=.002; Table 3).

**Table 3:** Comparison of mean Developmental Milestones Checklist (DMC III) scores between the intervention groups and the control group.

Score	Full package (n=307), mean (SD)	Control (n=199), mean (SD)	*P* value	Limited package (n=297), mean (SD)	Control (n=199), mean (SD)	*P* value
Fine motor	12.97 (4.80)	11.41 (5.09)	<.001^a^	12.59 (4.02)	11.41 (5.03)	<.001^a^
Gross motor	26.55 (9.52)	23.50 (10.60)	.56	27.95 (9.33)	23.50 (10.60)	<.001^a^
Language	13.57 (8.20)	13.68 (9.18)	.01^a^	17.75 (7.91)	13.68 (9.18)	<.001^a^
Total DMC III score	53.09 (20.02)	48.59 (23.32)	<.001^a^	58.29 (19.28)	48.59 (23.32)	.002^a^

^a^Significant results at *P*<.05.

### Comparison of Mean Developmental Milestone Scores Between the Full and Limited Package Groups

Overall, there was no statistical difference in developmental milestones between those who received the full package and those that received the limited package (*P*>.99; Table 4). However, the full package group had higher knowledge scores than the limited package group (9.02 vs 8.73; *P*=.048).

**Table 4:** Comparison of knowledge and mean Developmental Milestones Checklist (DMC III) scores between the full and limited package groups.

Score	Full package (n=307), mean (SD)	Limited package (n=297), mean (SD)	*P* value
Knowledge	9.02 (2.19)	8.73 (2.22)	.048^a^
Fine motor	12.97 (4.80)	12.59 (4.02)	.15
Gross motor	26.55 (9.52)	27.24 (9.44)	.97
Language	13.57 (8.20)	17.75 (7.91)	>.99
Total DMC III score	53.09 (20.02)	58.29 (19.28)	>.99

^a^Significant results at *P*<.05.

## Discussion

### Key Findings

In this study, using an interactive text messaging service to provide information and support to adolescent mothers improved their knowledge on nurturing infant care and the developmental milestones of their children at 1 year of age. Adding psychosocial support groups did not have superior impact on maternal knowledge nor infant developmental milestones compared with the interactive text messaging alone.

### Comparison With Other Studies

Other studies have showed mixed findings on the impact of providing information and psychosocial support to mothers; most showed improvement in some but not all of the growth and developmental parameters of the children. A cluster intervention trial in Zambia evaluating home visits and parenting groups found an improvement in some anthropometric measures (stunting) as well as language but no effects on motor skills, cognitive, or socio-emotional development [39]. In Uganda, an intervention to reduce maternal depression and improve child development through group education on feeding, hygiene, and stimulation improved the cognitive and language development of the children [40]. Other interventions in mothers that provide information and support have been shown to improve various nutritional parameters [41]. A randomized trial in Ethiopia found improvement in socio-emotional and language development after an intervention consisting of play stimulation during home visits [42]. However, a home-based parenting support program delivered until 6 months post partum in South Africa found no impact on the cognitive development of the infants [43]. Our study found improvements in both maternal knowledge and infant development parameters. One possible reason for this may be because we were intervening among adolescent mothers only, whereas the other studies were among mixed-age mothers. Adolescent mothers are at a very high risk of maternal depression due to the lack of familial social support and socioeconomic hardships; any intervention in a convenient approach therefore could have higher impact [44]. Most of the studies mentioned above had a component of home support visits; however, we found no added benefits of home visits or psychosocial support groups to either maternal knowledge or infant growth and development outcomes. A possible explanation is since we were using an interactive text messaging platform, the adolescent mothers were able to access both information and sense of support from the platform, similar to what they would have acquired through home visits or support groups, but with an added advantage of privacy and convenience. An expansion of such a platform to include peer-to-peer interaction can further changes, knowledge, and behavior on nurturing infant care [45]. Interactive mHealth solutions that give users the opportunity to ask question or seek clarifications are more acceptable and effective than one-way platforms and can increase access to health information, especially to special populations, in an equitable and cost-effective way [46-49]. This would be valuable in providing adolescent mothers with information and support on reproductive health, which are services traditionally offered at health facilities. Interestingly, ANC attendance was high for all the groups in this study; this implies that the main limiting factor may not be the lack of interaction with the health care system but the provision of tailored, adolescent-responsive reproductive health information conveniently.

### Limitations and Strengths

The findings of this study should be interpreted in consideration of some limitations. First, the study had a limited number of clusters. To minimize the likely effect on the type 1 error rate, the GEE model with a small-sample correction was performed. Due to reservations of community stakeholders on recruiting vulnerable adolescent mothers with no planned interventions, we were not able to recruit a control group at baseline. The control group was recruited during the end-line survey, in a different subcounty in the study county, among adolescent mothers with infants between the ages of 9-12 months (similar to the intervention groups at the end of follow-up). Therefore, uncontrolled differences between the intervention groups and the control group could have contributed, in part, to the observed differences in developmental outcomes. Studies with posttest-only controls also have a weakness in that they may not adequately measure the change brought about by maturation (threats to validity that happen over time during follow-up) or sensitization (the impact of the intervention groups being exposed to the survey at baseline) [36]. Additionally, the groups had significant differences in socioeconomic variables at recruitment, which could have influenced the outcomes due to residual confounding. Due to the COVID-19 pandemic, we were not able to conduct assessments at 6 and 12 months but had our final evaluation after 9 months of follow-up.

Our study also has several strengths. Our target population was a vulnerable segment of the female reproductive population, which is a leading contributor to maternal and infant morbidity as well as mortality. Since the lack of information on reproductive health among adolescents is a key causative factor, out study provides evidence on how such information and support can be availed efficiently to this vulnerable demographic. Second, other than the information itself, our implementation approach was to use currently available structures (mobile phones and community health strategy) to make available information and support to adolescent mothers. Only 5.8% (49/840) of eligible participants were excluded due to the lack of a reliable mobile phone. This can make scaling up both feasible and sustainable. The mHealth strategic framework and electronic community health information system by the Kenya Ministry of Health are some of the pathways through which our findings can be adopted into policy, to drive interventions targeted at adolescent mothers and their children.

### Conclusion

In this study, an interactive text messaging platform among adolescent mothers in rural Kenya improved both the knowledge of mothers on infant nurturing care and the development milestones of their infants. These findings, if replicated in other studies in different settings, can provide a mechanism of improving the overall reproductive health of adolescents in LMICs. Although the addition of support groups in such interventions has been adopted before, we did not find any additional benefit in improving developmental outcomes. Therefore, an interactive mHealth solution could serve as a minimum intervention package among this vulnerable group to improve health outcomes.

## Appendix

Multimedia Appendix 1 CONSORT-eHEALTH checklist (V 1.6.1).

## Abbreviations

ANC: antenatal care
DMC III: Developmental Milestones Checklist
GEE: generalized estimating equations
LMIC: low- and middle-income country
mHealth: mobile health
WHO: World Health Organization
